# Hot air stream reduces cytotoxicity of light-cured calcium hydroxide based cements

**DOI:** 10.4317/jced.56590

**Published:** 2020-03-01

**Authors:** Celso-Afonso Klein-Junior, Roberto Zimmer, Diana-Lina-Bronca Borghetti, Fernando-Freitas Portella, Flávia-Carolina Abich, Daniel-Rodrigo Marinowic, Keiichi Hosaka, Eduardo-Galia Reston

**Affiliations:** 1 Department of Operative Dentistry, School of Dentistry, Lutheran University of Brazil (ULBRA), Cachoeira do Sul, Rio Grande do Sul, Brazil; 2 Department of Operative Dentistry, School of Dentistry, Lutheran University of Brazil (ULBRA), Canoas, Rio Grande do Sul, Brazil; 3 Institute of Health Sciences, Universidade Feevale, ERS-239 2755, Novo Hamburgo, Rio Grande do Sul, Brazil; 4 Neuroscience Department, Brain Institute, Neuroscience Laboratory, Pontifical Catholic University of Rio Grande do Sul, Rio Grande do Sul, Brazil; 5 Department of Oral Health Science, School of Medical and Dental Science, Tokyo Medical and Dental University, Tokyo, Japan

## Abstract

**Background:**

The light-cured calcium hydroxide based cements have incomplete polymerization and unconverted monomers can cause pulp cell damage. The aim of this study was to evaluate the influence of a warm and hot air stream on the cytotoxicity of light-cured calcium hydroxide based cements.

**Material and Methods:**

The materials Dycal (conventional cement), Biocal, Hidrox-Cal, and Ultra-Blend Plus (light-cured calcium hydroxide cements) were submitted to cytotoxicity analysis after polymerization, without vs. with previous heat treatment with a warm (37°C) and a hot (60°C) air stream. Following polymerization, cements were maintained in culture medium for 24 hours and 7 days, and subjected to the MTT test. Data were analyzed using analysis of variance (ANOVA) followed by post-hoc Student-Newman-Keuls (**<0.05).

**Results:**

The results indicated significant differences between the materials according to their composition, i.e., light-cured cements treated with a jet of warm air showed similar cytotoxicity levels to those observed for conventional cement, suggesting that they may be considered alternatives in cases requiring pulp-capping treatment.

**Conclusions:**

Application of a hot air stream reduced cytotoxicity of materials tested.

** Key words:**Dental pulp capping, dental cements, calcium hydroxide, cell survival.

## Introduction

First introduced in Germany in 1920, calcium hydroxide has been the biomaterial most extensively studied over the years, with favorable clinical results (1). Calcium hydroxide may be used directly on pulp tissues or indirectly as pulp-capping material (2). It has a long history of clinical success and continues to be the gold standard when the goal is to protect the dentin-pulp complex (3,4). The main advantage of calcium hydroxide is its alkaline pH, as it creates an optimal setting for the stimulation of alkaline phosphatase, essential to the formation of mineralized barrier (5).

From a different standpoint, calcium hydroxide presents a low modulus of elasticity and low resistance to compression, which restrict its use in thin layers, in specific areas (6). The limited mechanical properties of chemically activated calcium hydroxide, combined with the requirement to accommodate the material in regions with the eminence of pulp exposure, has resulted in the development of light-curable calcium hydroxide based cements, composed of methacrylate monomers, with the following advantages: increased resistance, low solubility in acid settings, controlled working time, and achievement of optimal mechanical properties immediately after the light-curing process (7).

However, when resin materials are applied to deep cavities, diffusion of unpolymerized monomers into pulp tissues may occur through the dentinal tubules (8-10) and cause toxic effects (11-13). This situation can become more severe when the monomers come into direct contact with the dental pulp (14). Therefore, adequate conversion of monomers into polymers is essential to maximize the physical properties and clinical performance of light-curable calcium hydroxide based cements, as well as to reduce their cytotoxicity (15). Among several existing strategies to enhance polymerization rates, the use of high temperatures either before or during polymerization stands out (16-18). Several authors have demonstrated higher polymerization rates and therefore superior physical (16,19) and biological (20) properties of resin materials after the use of light-curing methods employing heat.

A fundamental feature for these materials is biocompatibility (21) and the first step in assessing biocompatibility is through cytotoxicity analysis based on cellular behavior and viability (22). Methacrylate-based dental materials are known to present a high level of cytotoxicity, and are therefore likely to penetrate the pulp and induce cytotoxic effects (23). Therefore, the aim of this study was to evaluate the influence of a warm and hot air stream on the cytotoxicity of light-cured calcium hydroxide cements.

## Material and Methods

-Sample preparation 

A conventional cement - Dycal (Dentsply - Milford, DE, USA) and three light-cured calcium hydroxide based cements: Biocal (Biodinâmica, Ibiporã, PR, Brazil), Hidrox-Cal (Maquira, Maringá, PR, Brazil), Ultra-Blend Plus (Ultradent Products Inc., South Jordan, UT, USA) were used in this *in vitro* study (Table 1). Conventional cement was mixed by 10s before heat treatment. All cements were treated in three different forms: 1) no heat treatment; 2) treatment with a jet of warm air (37°C); 3) treatment with a jet of hot air (60ºC). The jet air application lasted 5 seconds, distant 10 cm from the slide. All light-curable specimens were light-cured for 20 seconds using a VALO cordless light-emitting diode (LED) curing unit (Ultradent, Salt Lake City, UT, USA). The samples measuring 9 mm diameter x 1 mm thickness were prepared and immediately sterilized with ethylene oxide (Esteriliplus, Porto Alegre, RS, Brazil), (Table 1).

**Table 1:**
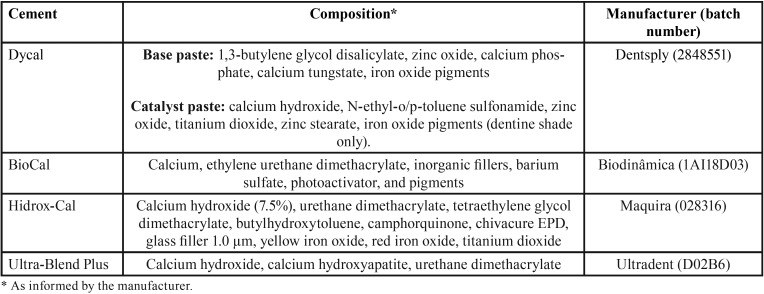
Calcium hydroxide based cement used, composition, batch, and manufacturer.

-Cell culture

The cells used in this study were NIH/3T3 mouse fibroblasts (ATCC®-American Type Culture Collection-TCC, Old Town, MD, USA) cultured in Dulbecco’s Modified Eagle Media (DMEM; Invitrogen®, Carlsbad, CA, USA). This medium was supplemented with 10% of fetal bovine serum, 100 U/mL of penicillin (Gibco), 100 U/mL of streptomycin (Gibco), and 100 μg/mL of gentamycin (Gibco). Cells were kept in a humidified incubator at a temperature of 37°C and 5% of CO2.

-Culture medium

Immediately after the mixing/curing process, specimens from the four groups were immersed in the DMEM medium. The specimen surface area to medium volume ratio was 3 cm2/mL, according to ISO 10993-12 (24). Surface area was calculated based on the total dimensions of the specimen, disregarding porosity. Extracts were tested for cell viability after remaining 24 hours and 7 days in the incubator.

-Cytotoxicity assay

The MTT method (Sigma-Aldrich, St. Louis, MO, USA) was used to assess cytotoxicity using the same parameters described elsewhere31. Briefly, at each treatment time (24 hours and 7 days), the culture medium was removed and 10% of an MTT solution (5 mg/mL) in phosphate buffer solution was added to each well. Subsequently, cultures were incubated at 37°C, protected from light, until the presence of blue-to-purple formazan crystals was observed. For the solubilization of formazan crystals, 100 μL of dimethyl sulfoxide (DMSO) was added to each well, and absorbance was measured at 570 nm wavelength using a spectrophotometer and an ELISA microplate reader (Benchmark Microplate Reader, Bio-Rad Inc., Hercules, CA, USA). The percentage of viable cells was calculated and compared to the results obtained with the negative control (cells cultured in DMEM). The assay was validated using a positive toxicity control (cells treated with 2% sodium hypochlorite).

-Statistical analysis

The cytotoxicity of calcium hydroxide based cements without previous heat treatment vs. those treated with a warm and a hot air stream were compared in terms of cell viability rates in NIH/3T3 mouse fibroblast cultures using three-way analysis of variance (ANOVA) followed by the post-hoc Student-Newman-Keuls test. Examiners were blinded to group allocation. Results were expressed as mean and standard deviation. Significance was set at 5%.

## Results

Table 2 shows the cell viability results obtained for the four calcium hydroxide based materials. Application of a warm or hot air stream did not influence cell viability in the conventional calcium hydroxide cement Dycal, which presented higher viability rates than resin-modified materials. Conversely, in all resin-based calcium hydroxide materials, the hot air stream enhanced cell viability at both 24 hours and 7 days. All tested materials presented lower rates of cell viability at 7 days compared to 24 hours.

**Table 2:**
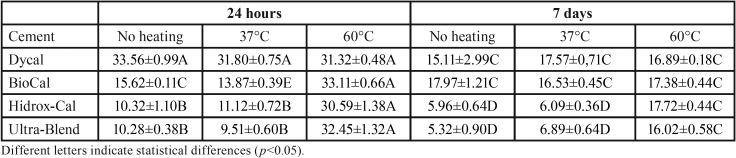
Mean ± standard deviation of cell viability rates (%) obtained at 24 hours and 7 days in light-cured and mixed calcium hydroxide based materials without and with previous heat treatment.

## Discussion

Light-curing pulp-capping materials were developed with the goal of simplifying the operating technique by facilitating material application. Due to their resin base, these materials are associated with increased mechanical resistance (4) and improved marginal seal as a result of lower dissolution rates (25). The present study showed that treatment of these materials with a jet of warm air just before their application resulted in cytotoxicity rates similar to those reported for chemically activated cements, most widely used at present. However, studies have shown that these cements, even when used for pulp capping, present a certain degree of cytotoxicity – a fact that becomes extremely relevant, as these cements are often used very close to dental pulp tissues.

The cytotoxicity associated with light-curing calcium hydroxide based cements and dentin adhesives is possibly a result of residual monomers (8). The monomer to polymer conversion rate for this type of material (dimethacrylate-based) is approximately 70%. Taking into consideration the proportion of monomers not completely converted and their possible diffusion into pulp tissues, it has been estimated that around 9% may leach when in contact with fluids (4). Therefore, the use of heat treatment before application of these materials has been investigated in an attempt to increase conversion rates and reduce the number of free monomers, and consequently the cytotoxic potential of the materials (20). The higher polymerization rates obtained as a result of heat treatment are explained by the increased mobility of monomers when heated (19).

In this study, treatment with a hot air stream (60°C) yielded significantly increased cell viability rates in methacrylate resin-based calcium hydroxide cements, both at 24 hours and at 7 days in comparison with the specimens that were not subjected to heating and with those treated with a jet of warm rather than hot air (37°C). In addition, the cell viability results observed in the two latter groups (no heating and heating to 37°C) was not different that the results observed for Dycal. Conventional cements have salicylate resin in their composition, and chemical curing occurs through chelation of calcium ions by the salicylate group; therefore, cell viability results in our conventional cement Dycal were not influenced by heat treatment, but it does influence monomer conversion in methacrylate resin-based cements. This explains the results obtained in the present study with BioCal, Hidrox-Cal, and Ultra-Blend Plus, which contain a urethane dimethacrylate-based matrix in their composition.

Cell viability results in the present study were similar to those found by the same authors in a previous study designed to assess self-adhesive resin cements (21). Also in that study, heat treatment to 60°C increased cell viability of fibroblasts, but viability levels reduced after 7 days. It is likely that a longer extraction time causes a larger number of unpolymerized monomers to leach from the polymerized resin matrix and provoke cell non-viability. Still, the level of cytotoxicity observed seems to be compatible with that observed in other materials with adequate clinical performance (26).

As already mentioned, the increased cell viability in dental materials is probably associated with an increased monomer-to-polymer conversion rate (20). However, in the present study, conversion rates were not measured; rather, cell viability essays were used, and viability was considered as a proxy for the effectiveness of the polymerization process. Other factors that could interfere with the biocompatibility of pulp-capping materials, e.g., release of calcium ions, alkalizing potential, solubility and antibacterial activity (27-29) should be explored in future studies.

In general, heat treatment through application of a hot air stream at 60°C before light-curing calcium hydroxide based cements reduced the cytotoxicity of materials. The adoption of this strategy as routine is justified in treatments that require pulp-capping, especially as it does not require an extra clinical step.

## References

[1] Da Rosa WLO, Cocco AR, Silva TM, Mesquita LC, Galarça AD, Silva AF (2018). Current trends and future perspectives of dental pulp capping materials: A systematic review. J Biomed Mater Res B Appl Biomater.

[2] Nilsen BW, Jensen E, Ortengren U, Michelsen VB (2017). Analysis of organic components in resin-modified pulp capping materials: critical considerations. Eur J Oral Sci.

[3] Komabayashi T, Zhu Q, Eberhart R, Imai Y (2016). Current status of direct pulp-capping materials for permanent teeth. Dent Mater J.

[4] Chen L, Suh BI (2017). Cytotoxicity and biocompatibility of resin-free and resin-modified direct pulp capping materials: A state-of-the-art review. Dent Mater J.

[5] Margunato S, Taşlı PN, Aydın S, Karapınar Kazandağ M, Şahin F (2015). In Vitro Evaluation of ProRoot MTA, Biodentine, and MM-MTA on Human Alveolar Bone Marrow Stem Cells in Terms of Biocompatibility and Mineralization. J Endod.

[6] Tam LE, Pulver E, McComb D, Smith DC (1989). Physical properties of calcium hydroxide and glass-ionomer base and lining materials. Dent Mater.

[7] Stanley HR, Pameijer CH (1985). Pulp capping with a new visible light-curing calcium hydroxide composition (Prisma VLC Dycal). Oper Dent.

[8] Leite MLAS, de Souza Costa CA, Duarte RM, Andrade AKM, Soares DG (2018). Bond Strength and Cytotoxicity of a Universal Adhesive According to the Hybridization Strategies to Dentin. Braz Dent J.

[9] Putzeys E, Duca RC, Coppens L, Vanoirbeek J, Godderis L, Van Meerbeek B (2018). In-vitro transdentinal diffusion of monomers from adhesives. J Dent.

[10] Cadenaro M, Maravic T, Comba A, Mazzoni A, Fanfoni L, Hilton T (2019). The role of polymerization in adhesive dentistry. Dent Mater.

[11] de Souza Costa CA, do Nascimento AB, Teixeira HM (2002). Response of human pulps following acid conditioning and application of a bonding agent in deep cavities. Dent Mater.

[12] de Souza Costa CA, Hebling J, Scheffel DL, Soares DG, Basso FG, Ribeiro AP (2014). Methods to evaluate and strategies to improve the biocompatibility of dental materials and operative techniques. Dent Mater.

[13] Bakopoulou A, Mourelatos D, Tsiftsoglou AS, Giassin NP, Mioglou E, Garefis P (2009). Genotoxic and cytotoxic effects of different types of dental cement on normal cultured human lymphocytes. Mutat Res.

[14] Hebling J, Giro EMA, de Souza Costa CA (1999). Biocompatibility of an adhesive system applied to exposed human dental pulp. J Endod.

[15] Ergun G, Egilmez F, Yilmaz S (2011). Effect or reduced exposure time on the cytotoxicity of resin luting cements cured by high-power led. J Appl Oral Sci.

[16] Lovell LG, Lu H, Elliott JE, Stansbury JW, Bowman CN (2001). The effect of cure rate on the mechanical properties of dental resins. Dent Mater.

[17] Daronch M, Rueggeberg FA, De Goes MF (2005). Monomer conversion of pre-heated composite. J Dent Res.

[18] Daronch M, Rueggeberg FA, De Goes MF, Giudici R (2006). Polymerization kinetics of pre-heated composite. J Dent Res.

[19] Klein-Jr CA, Zander-Grande C, Amaral R, Stanislawczuk R, Garcia EJ, Loguercio AD (2008). Evaporating solvents with a warm air-stream: Effects on adhesive layer properties and resin-dentin bond strengths. J Dent.

[20] Klein-Júnior CA, Zimmer R, Hentschke GS, Machado DC, Santos RB, Reston EG (2018). Effect of heat treatment on cytotoxicity of self-adhesive resin cements: Cell viability analysis. Eur J Dent.

[21] Mantellini MG, Botero TM, Yaman P, Dennison JB, Hanks CT, Nor JE (2003). Adhesive resin induces apoptosis and cell-cycle arrest of pulp cells. J Dent Res.

[22] Stanley HR (1992). Biological evaluation of dental materials. Int Dent J.

[23] Al Fawaz A, Gerzina TM, Hume WR (1993). Movement of resin cement components through acid-treated dentin during crown cementation in vitro. J Endod.

[24] (2002). Biological Evaluation of Medical Devices. Part 12: Sample Preparation and Reference Materials.

[25] Gopika GJ, Ramarao S, Usha C, John BM, Vezhavendhan N (2017). Histological evaluation of human pulp capped with light-cured calcium based cements: a randomized controlled clinical trial. Int J Sci Rep.

[26] Wegehaupt FJ, Lunghi N, Belibasakis GN, Attin T (2017). Influence of light-curing distance on degree of conversion and cytotoxicity of etch-and-rinse and self-etch adhesives. BMC Oral Health.

[27] Poggio C, Ceci M, Dagna A, Beltrami R, Colombo M, Chiesa M (2015). In vitro cytotoxicity evaluation of different pulp capping materials. Arh Hig Rada Toksikol.

[28] Luczaj-Cepowicz E, Marczuk-Kolada G, Pawinska M, Obidzinska M, Holownia A (2017). Evaluation of cytotoxicity and pH changes generated by various dental pulp capping materials - an in vitro study. Folia Histochem Cytobiol.

[29] Poggio C, Beltrami R, Colombo C, Ceci M, Dagna A, Chiesa M (2015). In vitro antibacterial activity of different pulp capping materials. J Clin Exp Dent.

